# Reducing neonatal infections in south and south central Vietnam: the views of healthcare providers

**DOI:** 10.1186/1471-2431-13-51

**Published:** 2013-04-09

**Authors:** Daniele Trevisanuto, Gaston Arnolda, Tran Dinh Chien, Ngo Minh Xuan, Le Thi Anh Thu, Danica Kumara, Ornella Lincetto, Luciano Moccia

**Affiliations:** 1 Children and Women’s Health Department, Medical School, University of Padua, Azienda Ospedaliera Padova, Via Giustiniani, 3, 35128 Padua, Italy; 2 Amici della Neonatologia Trentina, Trento, Italy; 3 East Meets West Foundation, Oakland, CA, USA; 4 Tu Du Hospital, Ho Chi Minh City, Vietnam; 5 Cho Ray Hospital, Ho Chi Minh City, Vietnam; 6 World Health Organization, Country Office for Vietnam, Hanoi, Vietnam

**Keywords:** Healthcare provider, Infection prevention, Low-resource country, Neonate

## Abstract

**Background:**

Infection causes neonatal mortality in both high and low income countries. While simple interventions to prevent neonatal infection are available, they are often poorly understood and implemented by clinicians. A basic understanding of healthcare providers' perceptions of infection control provides a platform for improving current practices. Our aim was to explore the views of healthcare providers in provincial hospitals in south and south central Vietnam to inform the design of programmes to improve neonatal infection prevention and control.

**Methods:**

All fifty-four participants who attended a workshop on infection prevention and control were asked to complete an anonymous, written questionnaire identifying their priorities for improving neonatal infection prevention and control in provincial hospitals in south and south central Vietnam.

**Results:**

Hand washing, exclusive breastfeeding and safe disposal of medical waste were nominated by most participants as priorities for preventing neonatal infections. Education through instructional posters and written guidelines, family contact, kangaroo-mother-care, limitation of invasive procedures and screening for maternal GBS infection were advocated by a smaller proportion of participants.

**Conclusions:**

The opinions of neonatal healthcare providers at the workshop accurately reflect some of the current international recommendations for infection prevention. However, other important recommendations were not commonly identified by participants and need to be reinforced. Our results will be used to design interventions to improve infection prevention in Vietnam, and may be relevant to other low-resource countries.

## Background

Globally, neonatal deaths (under 28 days of age) account for 43% of all deaths in children younger than 5 years of age [1]; ranging from 29% in Africa to 54% in Southeast Asia.

Three major causes of neonatal deaths account for more than 80% of all neonatal deaths globally: infections; complications of preterm birth; and intrapartum-related neonatal deaths (“birth asphyxia”) [1,2]. The relative contributions of these 3 causes of death vary both within and between countries [2], and are in part reflected by differences in the neonatal mortality rate (NMR – neonatal deaths per 1,000 live births). In settings with very high neonatal mortality rate (NMR > 45), about half the neonatal deaths are caused by infections [3]. In low-mortality settings (NMR < 15), only 15% of deaths are caused by infections, these deaths are more likely to take place in hygienic settings with access to antibiotics, and preventing them is more complex [2,3].

Vietnam has shown a strong commitment to achieving the fourth Millennium Development Goal (MDG 4) of reducing its under-five year mortality rate (U5MR) by two-thirds between 1990 and 2015. As a result, the U5MR and the infant mortality rate (IMR) have reduced [4]; the U5MR has reduced from 55⁄ 1,000 live births in 1990 to 24⁄ 1,000 live births in 2009 and the IMR has reduced from 39/1,000 live births to 20/1,000 live births [5]. By 2009 the NMR (12/1,000) accounts for half the U5MR (5), so the NMR will have to be reduced if Vietnam is to meet its MDG4 target of 18.3/1,000.

Facility-based neonatal care in Vietnam was very limited at the start of the 21^st^ century, and generally provided by non-specialist paediatricians in the paediatric wards. In under ten years newborn care has been rapidly expanded and today all provincial hospitals have neonatal units, usually in a specially designated area of the obstetric or paediatric wards, with staff drawn from the Pediatric Department. These units are usually referred to as Neonatal Intensive Care Units (NICUs), but relatively few have the capacity to provide long-term mechanical ventilation, so this paper refers to them as nurseries. At a minimum, these nurseries are equipped with CPAP, oxygen, warming devices, phototherapy and essential medicines and supplies to deal with the most common neonatal conditions. As an estimated 64% of deliveries are in institutions, and 88% are attended by a skilled attendant [5], a strong network exists for appropriate referral of sick neonates.

We are not aware of any data on the incidence of early- and late-onset neonatal sepsis in Vietnam as a whole, or specifically in the central or south central regions. In a province in northern Vietnam, however, a recent study based on verbal autopsy found that 59% of neonatal deaths occurred in the first 24 hours after birth [6]. In this study, infections could only be assigned as the cause of death in term infants, and was responsible for 11% of neonatal deaths. Preterm birth (<37 weeks gestation) was responsible for 40% of deaths, but it is unclear what proportion of these deaths had infection as their proximal cause.

The aim of our study was to explore the views of healthcare providers in provincial hospitals in south and south central Vietnam on how to improve neonatal infection prevention and control.

## Methods

A one-day workshop on “Infection prevention and control in the NICU” was held in Ho Chi Minh City (Vietnam) on 2^nd^ December, 2011. The workshop was organized by a non governmental organization (East Meets West Foundation, EMW), in collaboration with the World Health Organization (WHO), Hanoi, and an Italian Non-Government Organization Amici della Neonatologia Trentina. Tu Du Hospital in Ho Chi Minh City is the obstetric tertiary referral centre for all provincial hospitals located in south and south central Vietnam, and provided clinical facilitators for the workshop.

Vietnam has 58 provinces and 5 municipalities, with provinces further divided into districts; Provincial hospitals function as tertiary referral centres for districts hospitals. The average hospital stay averages two days after normal delivery and four days after caesarean section. Since 2004, a Non-Government Organization called East Meets West (EMW) has been implementing projects to improve neonatal care in Vietnam through a national program (‘Breath of Life’) which has helped more than 200 hospitals establish neonatal nurseries by providing training, equipment and alcohol handrub.

One doctor and one nurse from each of 30 provincial-level hospital in south and south central Vietnam were invited to attend the workshop. The primary aim of the workshop was to update participant knowledge on neonatal infection prevention and control, to discuss programmatic implications of the current international recommendations and to provide a basis for prioritization of interventions in their local settings. There was no pre- or post-workshop assessment of attendee knowledge or skills.

At the beginning of the workshop, participants were invited to complete a written questionnaire, in Vietnamese, identifying the activities that they believed could significantly control and prevent infection in their hospital. The questionnaire was presented to the participants, before they were offered the opportunity to complete it; participation was voluntary.

The questionnaire was divided into three main sections: a) information about the participant and his/her hospital (city, sex, age, professional background, and institutional role); b) questions about diagnostic, therapeutic and administrative practices at the hospital which relate to prevention and management of neonatal infections; and c) questions about methods to improve hospital-based infection prevention. In sections b) and c), no distinction was made between systemic and localized infections. Section c) was divided in 7 sub-sections (education, personnel, environment, instruments, care of the newborn, family access to the nursery and contact with the infant, and prophylaxis and therapy), each of which included three to six issues which participants were asked to grade by importance as a measure to prevent or manage infection. Grading was on a four-point Likert scale: (i) very important; (ii) important; (iii) not important; and (iv) useless.

Participants were allowed 30 minutes to complete the questionnaire, they were instructed not to consult with their colleagues during this time. The responses were analyzed in the offices of EMW in Hanoi. Survey responses were entered into an spreadsheet (Microsoft Excel 2010) by two different clerks using the same template, at different times. The two files were compared and discrepant results were checked against original forms and corrected.

Descriptive data for 2010 (e.g., number of births, number of admissions to the nursery) from each of the hospitals was collected prior to the workshop. This data collection was done in the following way: each hospital director was contacted by phone, and following his/her agreement, a detailed list of questions were sent to the hospitals by email, fax or post. Each hospital completed the data forms using their official 2010 reports, and the completed response was returned to EMW.

### Statistical analysis

Data are presented as numbers and/or percentages, as appropriate. Fisher’s exact test was used for comparison between groups. Statistical analysis was performed using R2.12 software (R Foundation for Statistical Computing, Wien, Austria).

## Results

Of 60 invited participants from 30 hospitals, 54 (90%) individuals attended the workshop, including 21 physicians, 29 nurses and 4 midwives. All attendees consented to complete the questionnaire. Of the 54 participants, 45 (83%) were female; 3 (6%) were heads of department, 9 (17%) were heads of nursing, and 42 (78%) were general staff members. The median (range) age of participants was 36 years (21–54). The majority of staff worked in the paediatric ward (n = 32) or the neonatal ward (n = 12), but there were five participants from the obstetric ward and five from the hospital infection control department. The majority of participants (n = 47) had professional experience of over 2 years duration, two participants had 1–2 years experience, and three had less than 6 months experience.

Table 1 presents descriptive data about the 30 provincial level hospitals in south and south central Vietnam where participants attending the meeting were working. Most attendees worked in general hospitals (93%) and the median distance to the tertiary referral hospital (Children Hospital No.1, Ho Chi Minh City) was 215 km. A total of about 200,000 neonates were born in these hospitals during 2010. Mortality data are shown in Table 1 and refer to both inborn and outborn neonates; disaggregated data by inborn/outborn status, and data on age at death were not available. Sepsis was the primary diagnosis for about a quarter of deaths.

**Table 1:** Selected characteristics of the 30 provincial level hospitals (2010) where attendees were working at the time of the workshop

General hospitals, n (%)	28 (93%)
Distance (km) from regional referral hospital, median (range)	215 (40–950)
Number of beds, median (range)	690 (100–1,500)
Number of births, median (range)	6,545 (0–13,871)
Total number of doctors in the unit, median (range)	10 (2–46)
Total number of nurses in the unit, median (range)	25 (4–70)
Total number of different rooms in the unit, median (range)	3 (0–11)
Total number of beds in the unit, median (range)	20 (0–75)
Total number of patients/year, median (range)	1,019 (100–4,258)
Number of inborn patients admitted to the unit, median (range)	713 (0–4,200)
Number of out born patients admitted to the unit, median (range)	204 (40–1,200)
Number of deaths in the unit, median (range)	40 (0–147)
Number of deaths in unit attributed to sepsis, median (range)	12 (0–50)
Availability of alcohol gel for quick hand-cleansing, n (%)	22 (73%)

Table 2 presents information on diagnostic, therapeutic and administrative practices to prevent and manage neonatal infections in hospitals where participants attending the meeting were working. In general, participants considered facilities for biochemical and cultural diagnosis of infection to be adequate in all hospitals. Clinical evaluation was considered to be an appropriate method to diagnose neonatal infection by a large proportion of participants (78%), followed by biochemical examination using CRP (63%); a smaller proportion (40%) considered culture results to be appropriate. For suspected infection, about half the participants (49%) commence therapy with two antibiotics, with one-third (33%) using a single antibiotic; the choice is usually based on a local protocol (37%) or a collaborative team decision (38%). The costs of laboratory examinations are usually charged to the patient’s insurance. Most participants (89%) stated that their hospitals routinely hold meetings to discuss and monitor infection control procedures and trends.

**Table 2:** Diagnostic, therapeutic and administrative practices to manage neonatal infections in hospitals where participants were working at the time of the survey

**Question**	**Response**	**Percent**
1. In your opinion, which is the most appropriate method to detect neonatal infection in your department? (One or more):	History	48
	Clinical assessment	78
	Biochemical examination (CRP)	63
	Culture	46
	Environmental evaluation	24
2. Do you routinely perform blood cultures for neonatal sepsis in your department?	Yes	42
	No	58
3 Where are blood cultures analyzed?	In my hospital	100
	In another public hospital	0
	In a private laboratory	0
4. How long does it take to receive the C-reactive protein or white blood count analysis?	<3 hours	85
	3-6 hours	12
	6-12 hours	3
	>12 hours	0
5 How long does it take to receive the blood culture analysis?	2 days	14
	3-4 days	41
	5-6 days	24
	>6 days	21
6. Is an antibiogram usually included with the blood culture results?	Always	91
	Sometimes	9
	Never	0
7. Do admitted neonates routinely receive prophylactic antibiotics?	Always	15
	Only on positive history and signs	75
	Never	10
8. For admitted neonates, what is your antibiotic strategy prior to receiving blood culture analysis?	Start with single antibiotic	33
	Start with two antibiotics	49
	Start with three antibiotics	18
9. How do you choose the antibiotic(s)	Individual decision	25
	Department protocol	37
	Collaborative team decision	38
10. Who pays for analysis of the blood culture?	Hospital	8
	Patient’s family	9
	Patient’s insurance	79
	Other	4
11. Are there routine meetings to discuss/monitor infection control procedures and trends?	Yes	89
	No	9
	Not stated	2
12: If yes to Q11, how often are the meetings?	Weekly	12
	Monthly	33
	Quarterly	49
	Bi-annually	0
	Annually	4
	Not stated	2
13. If yes to Q11, who participates in these meetings?	Doctors only	6
	Nurses only	4
	Doctors and nurses (‘clinicians’)	27
	ID staff and/or administrators only	12
	Clinicians + ID staff &/or administrators	49
	Not stated	2

(data are presented as percentages).

Table 3 reports the proportion of respondents who consider the presented methods of improving neonatal infection prevention and control as being ‘very important’. Hand washing (78%), clean physical environments (88%), safe disposal of medical waste (86%), exclusive breastfeeding (89%), and appropriate use of antibiotics (77%) were the strategies scored as ‘very important’ by more than three-quarters of respondents. Education through instructional posters (29%) and written guidelines (49%), strict monitoring of staff activity (40%) and surfaces/equipment (48%), full enteral feeding (33%), kangaroo mother care (49%), limiting maternal contact (29%), screening for maternal GBS infection (25%) and appropriate use of antepartum antibiotics (39%) were scored as ‘very important’ by fewer than half of all respondents. Similar proportions of doctors and nurses scored each item as ‘very important’ with the exception of maternal education on hygienic contact with the infant (56% among doctors vs. 84% among nurses; p < 0.05) and, possibly, safe disposal of medical waste (72% among doctors vs. 94% among nurses; p = 0.08) and the doctor/patient ratio (78% among doctors vs. 49% among nurses; p = 0.07).

**Table 3:** Proportion of respondents rating each option for improving infection prevention and control as ‘very important’, overall and separately for doctors and nurses

		**Proportion considering each intervention to be ‘very important’**		
		**Doctors**	**Nurses**	**Overall**
**Education/Guidance for staff**	Clinical meetings	66.7	72.7	70.6
	Instructional posters	33.3	27.3	29.4
	Written guidelines	44.4	51.5	49.0
	Strict monitoring of daily staff activity	29.4	46.7	40.4
**Personnel**	Nurse/patient ratio	83.3	63.6	70.6
	Doctor/patient ratio	77.8	48.5	58.8
	Hand washing	83.3	90.9	78.4
**Environment**	Cleaning of physical environment	77.8	78.8	88.2
	Presence of hand washing stations	77.8	68.8	72.0
	Presence of antiseptic gel solutions	47.1	64.5	58.3
	Dedicated room for infected patients	50.0	66.7	60.4
	Safe disposal of medical waste	72.2	93.5	85.7
	Monitoring of hygiene of surfaces/equipment	47.1	48.4	47.9
**Instruments**	Use of clean instruments	83.3	65.6	72.0
	Dedicated equipment for each patient	66.7	75.0	72.0
**Care of the newborn**	Limitation of invasive procedures	50.0	56.7	54.2
	Full enteral feeding	35.3	31.0	32.6
	Exclusive breastfeeding	94.1	86.7	89.4
	Kangaroo mother care	52.9	46.7	48.9
**Family contacts and education**	Limitation of maternal visits	33.3	25.8	28.6
	Limitation of paternal visits	44.4	64.5	57.1
	Limitation of family member visits	38.9	58.1	51.0
	Maternal education on hygienic contact	55.6	83.9	73.5
	Family education on hygienic contact	55.6	71.0	65.3
	Pre-discharge education to prevent re-admission	64.7	77.4	72.9
**Prophylaxis and therapy**	Screening for maternal GBS	16.7	30.0	25.0
	Antepartum antibiotics for maternal infection	33.3	41.9	38.8
	Appropriate use of antibiotics	70.6	80.6	77.1

## Discussion

Severe infections represent the main cause of neonatal mortality, accounting for more than 1 million neonatal deaths worldwide every year [1], and is a common cause of neonatal mortality in both high- and low-resource settings [3,7,8]. This study asked healthcare providers from south and south central Vietnamese provincial hospitals about the importance they assign to selected strategies for improving neonatal infection prevention and control.

The selected strategies for improving neonatal infection prevention and control were scored as ‘very important’ or “important” by the large majority of participants, confirming their relevance to clinicians. Certain groups of strategies, however, were less frequently considered ‘very important’. For example, “Educational” strategies (in particular, instructional posters, written guidelines, and strict monitoring of daily staff activity) were less frequently considered “very important”. These data contrast with previous studies showing that “education of health staff” was scored as the highest priority by local healthcare providers for improving the delivery room setting and maternal and neonatal departments in low- and high-resource settings [9,10]. These differences may be attributable to differences in topic area (neonatal resuscitation vs. infection prevention) or by different backgrounds of participants. It is notable that other studies have found that the strategy of using instructional posters and written guidelines, recommended by several health agencies for educational purposes, doesn’t receive great support from healthcare providers [11].

Strategies relating to “Prophylaxis and therapy” (e.g., antepartum antibiotics for maternal infection or Premature Rupture of Membranes [PROM], and screening for maternal Group B Streptococcus [GBS] infection) were also less frequently considered “very important”. Screening for maternal GBS infection and antepartum antibiotics are recommended by international guidelines for reducing neonatal sepsis [12], and in high-resource settings, recommendations for antenatal universal screening for GBS have been rapidly adopted [13]. However, participants involved in this survey gave a low priority to these issues, and nurses/midwives appear less likely to consider these interventions ‘very important’ than doctors. We know of no national data on the prevalence of GBS infection in Vietnam; screening and intervention are not part of the national standard guidelines for antenatal care. In the United States, between 5% and 40% of all pregnant women have recto-vaginal colonization with GBS [14]. In Taiwan, a recent study found that the maternal colonization rate of GBS was around 20% at hospital base and the incidence of neonatal GBS infection was 1 per 1000 live births of infants born at hospitals. The authors concluded that “universal maternal recto-vaginal culture of GBS with intrapartum antibiotic prophylaxis is required to reduce early-onset disease and mortality because of GBS infection in neonates in Taiwan” [15]. The results of this survey suggest that advocacy of antenatal universal screening for GBS should be considered as part of future hospital intervention strategies, and at national policy level; such advocacy is dependent on the incidence of GBS infections in neonates which will determine, in large part, the cost-effectiveness of such a strategy [16].

Hand washing, cleaning the physical environment, safe disposal of medical waste and exclusive breastfeeding were the strategies most frequently scored as‘very important’ for preventing neonatal infections. The WHO strongly advocates hand hygiene and exclusive breastfeeding for preventing and reducing infections [11]. In Vietnam, extensive training has been conducted by the MOH and partners in the areas of hand hygiene (due to recent epidemics of SARS, avian influenza and H5N1 influenza), breastfeeding (ongoing MOH campaign since August 2011) and safe disposal (ongoing WHO program on safety). The lesson seems to be well learned by healthcare providers involved in the present survey. National data on breastfeeding in Vietnam, however, indicate that only 58% of neonates are breastfed early, and only 17% of infants are exclusively breastfeeding at 6 months, so community-wide promotion of breastfeeding may be required.

Improvement of nurse/patient and doctor/patient ratios for preventing infections were scored as ‘very important’ by 71% and 59% of participants respectively, reflecting the evidence that nurses, more than doctors, play an important role in this field [10,17]. Interestingly, however, a larger proportion of doctors than nurses rated the improvement of both these ratios as ‘very important’.

Cleaning of the physical environment was scored as ‘very important’ by 88% of participants, but the “Presence of antiseptic gel solutions” was considered ‘very important’ by only 58% of participants. The use of antiseptic gel solutions for infection prevention has been suggested [18,19], but its efficacy in low resource settings remains to be proved [20]. This information needs to be evaluated in depth because alcohol gel handrubs for quick hand hygiene were available in the majority of hospitals (73%) where participants were working; WHO Guidelines recommend routine use of alcohol-based handrubs as the gold standard in health care worldwide (after initial hand washing) [21].

Most of the strategies relating to “Care of the newborn” were considered as ‘very important’ by a relatively small percentage of participants; “Limitation of invasive procedures” (54%); “Full enteral feeding” (33%); and “Kangaroo-mother-care” (49%). In both high- and low-resource settings, limitation of invasive procedures and the early achievement of full enteral feeding are considered crucial to prevent and to limit neonatal infections [7,8,22]. Maternal involvement in the process of care, through kangaroo-mother-care, is suggested as a method of reducing neonatal mortality and preventing infections in stabilized low birthweight infants in low resources settings [23]. While Vietnamese mothers and family members are routinely involved in the care of the newborn, in part as a response to suboptimal nurse-patient ratios, the majority of participants scored as ‘very important’ the strategy of limiting paternal and family member visits for preventing neonatal infections, and 29% stated that limiting maternal visits was very important. The limited importance assigned by participants to maternal access and to kangaroo mother care needs to be explored further.

The judicious use of antibiotics may reduce the emergence of resistant bacterial strains and limit the side-effects of prolonged and unnecessary antibiotic courses. This important issue was reviewed by Isaacs in 2006, who suggested a ten point plan to reduce antibiotic resistance in neonatal units [7,8]. The appropriate use of antibiotics was scored as ‘very important’ by 77% of participants, but their antibiotic strategy prior to receiving blood culture results differed widely: one third start with a single antibiotic, half start with two antibiotics, and 18% with three antibiotics. In only 37% of the participating centres was the choice of antibiotic(s) based on a written departmental protocol. These inter-hospital variations suggest a need for higher-level guidelines [24].

The report of a conference on potential use of chlorhexidine in low-resource settings [25] notes that, studies of chlorhexidine have focused on three primary uses: a) intrapartum vaginal and neonatal wiping, b) neonatal wiping alone, and c) umbilical cord cleansing. Studies of chlorhexidine vaginal and infant wipes have not shown reductions in perinatal mortality and morbidity [26,27]. Data from three cluster-randomized trials, however, demonstrate that a single application of 4% chlorhexidine to the umbilical cord stump following delivery reduces the incidence of omphalitis and neonatal mortality, especially in preterm newborns [28]. This intervention, which is safe and inexpensive and requires minimal training and skill, should be considered for home births. The WHO currently recommends dry cord care for newborns [11], and this practice is the standard of care in Vietnamese hospitals. It remains to be demonstrated if the application of 4% chlorhexidine to the umbilical cord stump following hospital deliveries could be effective in improving neonatal outcomes.

Seventy-eight percent of attendees considered physical examination to be an appropriate method of detecting infection, while only 63% considered biochemical examination (CRP) to be an appropriate method, and 40% considered culture to be appropriate. The reasons behind these views were not formally investigated as part of this study. Our experience of working in low resource settings suggest a number of possibilities, including lack of 24-hour access to laboratory services and the frequent failure to successfully culture organisms from neonates who have clear signs of infection. In these circumstances, clinicians are forced to make a prompt diagnosis based on clinical signs and treat presumptively.

Some limits of this study need to be considered. This survey, while achieving near-complete coverage of the majority of provincial-level hospitals in south and south central Vietnam, was based on a convenience sample that included a limited number of participants with different professional backgrounds and roles. The majority of participants, however, were considered local experts in neonatal infections and had responsibilities for neonatal infection control in their hospitals. Their opinions are therefore highly relevant.

While information from this survey identifies issues which may need to be addressed during any hospital-based intervention, the final content of an intervention must be guided by the actual circumstances in the target hospitals. The descriptive data provided by each hospital as part of this study is too limited to provide a detailed prescription. A first step in any hospital where intervention is planned is likely to include early introduction of universal protocols for low-cost interventions such as initial hand washing and subsequent use of alcohol-based handrubs, and advocacy of early skin to skin contact and initiation of breastfeeding, while at the same time establishing data collection systems that can provide local epidemiological information about neonatal infectious disease.

## Conclusions

Our survey reveals that infections are reported to be a leading cause of neonatal mortality (an average of 25% of all cases) in provincial hospitals in south and south central Vietnam. Some strategies previously identified by international health agencies as milestones in the prevention of neonatal infections, such as hand washing, exclusive breastfeeding and safe disposal of medical waste, were rated as ‘very important’ by almost all local healthcare providers. Conversely, other issues deemed as relevant for infection prevention in low-resource settings, such as family contact, kangaroo-mother-care, limitation of invasive procedures, and screening for maternal GBS infection, appeared to be under-appreciated. Education through instructional posters and written guidelines, while strongly advocated by agencies involved in international programs in low-resource countries, was considered to be relatively unimportant in our sample. While there is a great need for continued research on cost-effectiveness of key interventions in setting with limited resources, understanding the prior opinions of clinicians involved in maternal and neonatal health can contribute to better design and implementation of intervention programs.

## Competing interests

GA and LM, have temporary professional contracts with the East Meets West Foundation, Oakland, California. OL is an employee at the World Health Organization, Country Office for Vietnam. TDC and DK are employees of the East Meets West Foundation, Oakland, California. NMX, LTAT and DT declare that they have no competing interests. No honorarium was received by all the authors for this article.

## Authors’ contributions

DT had primary responsibility for preparation of the questionnaire and writing the manuscript. GA and TDC collected and analyzed the data, and contributed to the writing. DK, NMX and LTAT contributed to the preparation of the questionnaire, involved participants in the project, helped in interpretation of the data and contributed to the writing. LM and OL supervised the design and execution of the study, contributed to the final data analyses and to the writing of the manuscript. All the authors read and approved the final manuscript. OL is a staff member of the WHO. The author alone is responsible for the views expressed in this publication and they do not necessarily represent the decisions or policies of the WHO.

## Pre-publication history

The pre-publication history for this paper can be accessed here:

http://www.biomedcentral.com/1471-2431/13/51/prepub

## References

[B1] Black RE Cousens S Johnson HL Lawn JE Rudan I Bassani DG Jha P Campbell H Walker CF Cibulskis R Eisele T Liu L Mathers C Child Health Epidemiology Reference Group of WHO and UNICEF Global, regional, and national causes of child mortality in 2008: a systematic analysis Lancet 2010 375 9739 1969 1987 20466419 10.1016/S0140-6736(10)60549-1

[B2] UNICEF, WHO, World Bank, UN Population Division Levels and trends in child mortality 2012 [http://www.who.int/maternal_child_adolescent/documents/levels_trends_child_mortality_2012.pdf]

[B3] Lawn JE Cousens S Zupan J Lancet neonatal survival steering team. 4 Million neonatal deaths: when? where? Why? Lancet 2005 365 9462 891 900 10.1016/S0140-6736(05)71048-5 15752534

[B4] Hoa DP Nga NT Malqvist M Persson LA Persistent neonatal mortality despite improved under-five survival: a retrospective cohort study in northern Vietnam Acta Paediatr 2008 97 2 166 170 10.1111/j.1651-2227.2007.00604.x 18254906

[B5] UNICEF The state of the world’s children 2011 2011 New York: UNICEF

[B6] Nga NT Hoa DT Målqvist M Persson LÅ Ewald U Causes of neonatal death: results from NeoKIP community-based trial in Quang Ninh province, Vietnam Acta Paediatr 2012 101 4 368 373 10.1111/j.1651-2227.2011.02513.x 22107114

[B7] Borghesi A Tzialla C Decembrino L Manzoni P Stronati M New possibilities of prevention of infection in the newborn J Maternal Fetal Neonatal Med 2011 24 Suppl 2 28 30 10.3109/14767058.2011.604934 21749301

[B8] Clark R Powers R White R Bloom B Sanchez P Benjamin DK Jr Nosocomial infection in the NICU: a medical complication or unavoidable problem? J Perinatol 2004 24 6 382 388 10.1038/sj.jp.7211120 15116140

[B9] Trevisanuto D Lincetto O Doglioni N Micaglio M Zanardo V Improving the delivery room setting in developing countries: the opinion of local health caregivers Acta Paediatr 2008 97 8 1045 1048 10.1111/j.1651-2227.2008.00820.x 18435814

[B10] Trevisanuto D Bavuusuren B Wickramasinghe CS Dharmaratne SM Doglioni N Giordan A Zanardo V Carlo W Improving maternal and neonatal departments in high and low resource settings: the opinion of local health providers J Matern Fetal Neonatal Med 2011 24 10 1267 1272 10.3109/14767058.2010.546911 21261448

[B11] World Health Organization Managing newborn problems: a guide for doctors, nurses, and midwives 2003 WHO Library Cataloguing-in-Publication Data

[B12] Verani JR McGee L Schrag SJ Division of Bacterial Diseases Prevention of perinatal group B streptococcal disease--revised guidelines from CDC, 2010 MMWR Recomm Rep 2010 59 RR-10 1 36 21088663

[B13] Van Dyke MK Phares CR Lynfield R Thomas AR Arnold KE Craig AS Mohle-Boetani J Gershman K Schaffner W Petit S Zansky SM Morin CA Spina NL Wymore K Harrison LH Shutt KA Bareta J Bulens SN Zell ER Schuchat A Schrag SJ Evaluation of universal antenatal screening for group B streptococcus N Engl J Med 2009 360 25 2626 2636 10.1056/NEJMoa0806820 19535801

[B14] Winn HN Group B streptococcus infection in pregnancy Clin Perinatol 2007 34 3 387 392 10.1016/j.clp.2007.03.012 17765489

[B15] Yu HW Lin HC Yang PH Hsu CH Hsieh WS Tsao LY Chen CH Lin HC Tseng YC Group B streptococcal infection in Taiwan: maternal colonization and neonatal infection Pediatr Neonatol 2011 52 4 190 195 10.1016/j.pedneo.2011.05.008 21835363

[B16] Schwab F Meyer E Geffers C Gastmeier P Understaffing, overcrowding, inappropriate nurse:ventilated patient ratio and nosocomial infections: which parameter is the best reflection of deficits? J Hosp Infect 2012 80 2 133 139 10.1016/j.jhin.2011.11.014 22188631

[B17] Baltimore RS Consequences of prophylaxis for group B Streptococcal infections of the neonate Semin Perinatol 2007 31 1 33 38 10.1053/j.semperi.2007.01.005 17317425

[B18] Boyce JM Pittet D Guideline for hand hygiene in health-care settings: recommendations of the Healthcare Infection Control Practices Advisory Committee and the HICPAC/SHEA/APIC/IDSA hand hygiene task force Infect Control Hosp Epidemiol 2002 23 12 suppl S3 S40 12515399 10.1086/503164

[B19] Widmer AE Danger M Alcohol-based handrub: evaluation of technique and microbiological efficacy with international infection control professionals Infect Control Hosp Epidemiol 2004 25 3 207 209 10.1086/502379 15061411

[B20] Gill CJ Mantaring JB Macleod WB Mendoza M Mendoza S Huskins WC Goldmann DA Hamer DH Impact of enhanced infection control at 2 neonatal intensive care units in the Philippines Clin Infect Dis 2009 48 1 13 21 10.1086/594120 19025496 PMC3866590

[B21] World Health Organization WHO guidelines on hand hygiene in health care. Geneva 2009 [http://whqlibdoc.who.int/publications/2009/9789241597906_eng.pdf]

[B22] Landre-Peigne C Ka AS Peigne V Bougere J Seye MN Imbert P Efficacy of an infection control programme in reducing nosocomial bloodstream infections in a Senegalese neonatal unit J Hosp Infect 2011 79 2 161 165 10.1016/j.jhin.2011.04.007 21820760

[B23] Conde-Agudelo A Belizán JM Diaz-Rossello J Kangaroo mother care to reduce morbidity and mortality in low birthweight infants Cochrane Database Syst Rev 2011 3 CD002771 11034759 10.1002/14651858.CD002771

[B24] Muller-Pebody B Johnson AP Heath PT Gilbert RE Henderson KL Sharland M iCAP Group (Improving Antibiotic Prescribing in Primary Care) Empirical treatment of neonatal sepsis: are the current guidelines adequate? Arch Dis Child Fetal Neonatal Ed 2011 96 1 F4 F8 10.1136/adc.2009.178483 20584804

[B25] McClure EM Goldenberg RL Brandes N Darmstadt GL Wright LL Armbruster D Biggar R Carpenter J Free MJ Mattison D Mathai M Moss N Mullany LC Schrag S Tielsch J Tolosa J Wall SN Schuchat A Smine A CHX Working Group The use of chlorhexidine to reduce maternal and neonatal mortality and morbidity in low-resource settings Int J Gynaecol Obstet 2007 97 2 89 94 10.1016/j.ijgo.2007.01.014 17399714 PMC2727736

[B26] Lumbiganon P Thinkhamrop J Thinkhamrop B Tolosa JE Vaginal chlorhexidine during labour for preventing maternal and neonatal infections (excluding Group B Streptococcal and HIV) Cochrane Database Syst Rev 2004 4 CD004070 15495077 10.1002/14651858.CD004070.pub2

[B27] Saleem S Rouse DJ McClure EM Zaidi A Reza T Yahya Y Memon IA Khan NH Memon G Soomro N Pasha O Wright LL Moore J Goldenberg RL Chlorhexidine vaginal and infant wipes to reduce perinatal mortality and morbidity: a randomized controlled trial Obstet Gynecol 2010 115 6 1225 1232 10.1097/AOG.0b013e3181e00ff0 20502294 PMC3893917

[B28] Goldenberg RL McClure EM Saleem S A review of studies with chlorhexidine applied directly to the umbilical cord Am J Perinatol 2012 Dec 19. [Epub ahead of print] 10.1055/s-0032-1329695 PMC3875170 23254380

